# The volume and the distribution of premorbid white matter hyperintensities: Impact on post‐stroke aphasia

**DOI:** 10.1002/hbm.26568

**Published:** 2024-01-15

**Authors:** Veronika Vadinova, A. J. Sihvonen, F. Wee, K. L. Garden, L. Ziraldo, T. Roxbury, K. O'Brien, D. A. Copland, K. L. McMahon, S. L. E. Brownsett

**Affiliations:** ^1^ Queensland Aphasia Research Centre University of Queensland Brisbane Australia; ^2^ School of Health and Rehabilitation Sciences University of Queensland Brisbane Australia; ^3^ Centre of Research Excellence in Aphasia Recovery and Rehabilitation La Trobe University Melbourne Australia; ^4^ Cognitive Brain Research Unit (CBRU) University of Helsinki Helsinki Finland; ^5^ Centre of Excellence in Music, Mind, Body and Brain University of Helsinki Helsinki Finland; ^6^ School of Clinical Sciences, Centre for Biomedical Technologies Queensland University of Technology Brisbane Australia

**Keywords:** aphasia, corpus callosum, language comprehension, stroke, white matter hyperintensities

## Abstract

White matter hyperintensities (WMH) are a radiological manifestation of progressive white matter integrity loss. The total volume and distribution of WMH within the corpus callosum have been associated with pathological cognitive ageing processes but have not been considered in relation to post‐stroke aphasia outcomes. We investigated the contribution of both the total volume of WMH, and the extent of WMH lesion load in the corpus callosum to the recovery of language after first‐ever stroke. Behavioural and neuroimaging data from individuals (*N* = 37) with a left‐hemisphere stroke were included at the early subacute stage of recovery. Spoken language comprehension and production abilities were assessed using word and sentence‐level tasks. Neuroimaging data was used to derive stroke lesion variables (volume and lesion load to language critical regions) and WMH variables (WMH volume and lesion load to three callosal segments). WMH volume did not predict variance in language measures, when considered together with stroke lesion and demographic variables. However, WMH lesion load in the forceps minor segment of the corpus callosum explained variance in early subacute comprehension abilities (*t* = −2.59, *p* = .01) together with corrected stroke lesion volume and socio‐demographic variables. Premorbid WMH lesions in the forceps minor were negatively associated with early subacute language comprehension after aphasic stroke. This negative impact of callosal WMH on language is consistent with converging evidence from pathological ageing suggesting that callosal WMH disrupt the neural networks supporting a range of cognitive functions.

## Abstract 2

We investigated the contribution of both the total volume of white matter hyperintensities (WMH), and the extent of WMH lesion load in the corpus callosum to the recovery of language comprehension and language production in aphasia after first‐ever stroke. WMH lesion load in the forceps minor, but not the total volume, was negatively associated with language comprehension after stroke. Frontal callosal WMH lesions may constitute a valuable cross‐diagnostic biomarker of poor cognitive outcomes after stroke.

## 1 INTRODUCTION

Post‐stroke aphasia, a language impairment that affects approximately a third of stroke survivors (Code & Petheram, 2011; Wu et al., 2020), is among the most debilitating cognitive consequences of stroke (Berthier, 2005; Gottesman & Hillis, 2010). While research has shown that multiple neurological, demographic and health‐related factors explain some of the variability in language recovery (Johnson et al., 2022; O'Sullivan et al., 2019; Watila & Balarabe, 2015), they do not reliably account for the spectrum of clinical recovery observed. In some individuals with stroke, the quantifiable status of pre‐stroke brain tissue integrity may serve as an indicator of susceptibility to more severe impairments, or to diminished recovery potential in the presence of stroke pathology (Appleton et al., 2020; Evans et al., 2022; Umarova, 2017). For example, it has been shown that characteristics of the spared brain tissue such as an increased total brain age (Kristinsson et al., 2022), a decreased hippocampal volume (Schevenels et al., 2022) or a high number of hyperintense vessels, a marker of abnormal hemodynamic function (Bunker et al., 2022), are negatively associated with language recovery in post‐stroke aphasia. These studies corroborate that inter‐individual variation in at least some neuroimaging markers that quantify common brain alterations in ageing are associated with functional stroke outcomes and can therefore be included in stroke prediction models. The most prevalent aging pathology of presumed vascular origin, white matter hyperintensities (WMH), has been identified as a promising proxy measure of cognitive outcomes in ageing and in disease (Prins & Scheltens, 2015).

WMH are a radiological marker associated with ageing and cerebrovascular risk factors (Debette et al., 2018; Grosu et al., 2021; Wardlaw et al., 2013) that have been negatively associated with cognitive skills (Camerino et al., 2020; Duering et al., 2014; Hamilton et al., 2021), including those skills requiring language in healthy individuals (Hilal et al., 2021; Jiang et al., 2018) and individuals with mild cognitive impairment (Jiang et al., 2018). Given that the incidence of WMH increases with age (Longstreth Jr. et al., 1996), they often coincide with aging‐associated cerebral pathologies, for example, stroke (Georgakis et al., 2019; Kim et al., 2020; Schwartz et al., 2018). A recent meta‐analysis of over 100 studies showed that the presence and the severity of premorbid WMH lesions in individuals with stroke is associated with an increased risk of dementia, functional impairment, stroke recurrence, and mortality after stroke (Georgakis et al., 2019). Fewer studies (*N* = 5) have examined the relationship between WMH burden and aphasia outcomes after stroke. Wright et al. (2018), Johnson et al. (2022), and Vadinova et al. (2023) identified a negative association between the degree of WMH and aphasia outcomes at different stages of recovery. Varkanitsa et al. (2020) also found that a greater amount of WMH is negatively associated with response to language therapy, and a study by Basilakos et al. (2019) also showed that a greater amount of WMH predicts a more rapid decline in language skills in chronic aphasia. Together, these studies suggest that WMH severity, assessed on clinical severity scales [e.g., Fazekas scale (Fazekas et al., 1987); Cardiovascular Health Study scale (Manolio et al., 1994)], could represent a clinically meaningful risk factor for poor language outcomes after stroke.

While WMH burden is assessed in clinical practice using qualitative severity scales (Fazekas et al., 1987; Manolio et al., 1994; Scheltens et al., 1993), WMH can also be assessed quantitatively by considering their volume (Hairu et al., 2021; Silbert et al., 2008) and anatomical distribution (Clancy et al., 2022; Hawe et al., 2018). Quantitative WMH measures have the potential to produce more precise and systematic assessment of observed WMH radiological lesions within the entire brain or within specific white matter tracts (Camerino et al., 2020; Duering et al., 2014). Similar to other cerebral lesions (e.g., stroke, multiple sclerosis), the quantitative assessment of WMH lesions (i.e., volume, anatomical distribution) has revealed associations between cardiovascular pathology and cognition, and the variance in cognitive outcomes in stroke cohorts (Bonkhoff et al., 2022; Clancy et al., 2022; Hawe et al., 2018; Röhrig et al., 2022). With respect to the critical role of WMH spatial distribution, converging evidence from lesion‐symptom mapping (LSM) studies have shown that some white matter tracts, such as the thalamic radiations and corpus callosum (CC) segments, are most commonly associated with cognitive deficits when affected by WMH (Biesbroek et al., 2016; Biesbroek et al., 2020; Camerino et al., 2020; Duering et al., 2011; Duering et al., 2014; Hilal et al., 2021; Jiang et al., 2018; Lampe et al., 2019; Zhao et al., 2018) (for a review, see Biesbroek et al., 2017). More recent studies specifically investigating the role of WMH lesion loads within the CC provide further evidence that callosal WMH lesions are independently associated with cognitive impairment in individuals with VCI and healthy ageing individuals (Freeze et al., 2022; Vemuri et al., 2021), suggesting that callosal connections are a strategic location within the white matter network.

The CC is a major interhemispheric tract supporting several critical functions, including excitation, inhibition and modulation of neuronal activity (for a review, see Bloom & Hynd, 2005; Innocenti et al., 2022). It is therefore not surprising that damage to this tract can impair any of these critical functions. Callosal connections are not considered to form part of the core left‐lateralised language network (Hagoort, 2014; Hickok & Poeppel, 2004; Price, 2012; Saur et al., 2008; Vigneau et al., 2011). However, given that the right hemisphere (RH) has been shown to contribute to language function when the primary network is perturbed (Brownsett et al., 2014; Geranmayeh et al., 2017; Schneider et al., 2022), communication between the hemispheres most likely occurs via the interhemispheric white matter tracts such as the CC, and others. While the precise contribution of the RH in language recovery remains unclear and unspecified (for a review, see Gainotti, 2015; Turkeltaub, 2015), neuroimaging evidence suggests that either language homologues and/or RH nodes within domain‐general networks play a role in supporting language recovery (Brownsett et al., 2014; Chang & Lambon Ralph, 2020; Geranmayeh et al., 2017; Hope et al., 2017; Stefaniak et al., 2022; Xing et al., 2016). Therefore, interhemispheric connections, such as the CC, may play a vital role in enabling RH compensation or upregulation in aphasia. WMH within these structures may impact on the effectiveness of compensatory or upregulatory processes reliant on these connections. Given this potential role of interhemispheric connections in enabling RH engagement during language recovery processes (Brownsett et al., 2014; Geranmayeh et al., 2017; Stefaniak et al., 2022), the contribution of callosal WMH to aphasia recovery warrants further investigation.

### 1.1 Present study

In this study, we investigated quantitative measures of early subacute WMH lesions to see if they explained variation in aphasia outcomes after stroke. We then considered if these measures could serve as a surrogate measure of pathological processes contributing to diminished structural brain integrity impacting recovery potential in aphasia. WHM burden has been shown to increase slowly after stroke (Clancy et al., 2022). A recent meta‐analysis found an average progression of 1.74 ml in WMH volume over 2.7 years (Jochems et al., 2022). In our study, the WMH was measured on average 27 days after the stroke, and so increases in WMH burden from pre‐stroke levels would be negligible. As a result, we propose that early subacute WMH burden, acquired within 6 weeks of the stroke event, reflects premorbid WMH levels. We considered the contribution to spoken comprehension and production outcomes of the total WMH volume and the WMH lesion load within callosal segments including, forceps minor (CC‐Fmin), forceps major (CC‐Fmaj) and the body (CC‐Body). We hypothesized that volumetric assessment would explain a proportion of variation observed in post‐stroke aphasia recovery. Second, we hypothesized that WMH lesion load within callosal segments would be negatively associated with language outcomes in aphasia.

## 2 METHODS

### 2.1 Participants

This study retrospectively analysed data from two post‐stroke aphasia studies. Inclusion criteria were (a) a single left‐hemisphere stroke (ischaemic or haemorrhagic), confirmed on the radiologist report, (b) the presence of aphasia, diagnosed using the WAB (Kertesz & Raven, 2007), (c) English as primary language, (d) availability for an initial assessment at 2–6 weeks post‐stroke onset, and (e) able to provide informed consent. Exclusion criteria were (a) history of neurological disorder, mental illness, head trauma, alcoholism, or cerebral tumour, (b) contraindications to magnetic resonance imaging (MRI), (c) severity of deficits precluding informed consent, (d) severe dysarthria or apraxia of speech (determined by a speech pathologist), and (e) severe hearing impairment. Apraxia of speech was assessed on the Apraxia Battery for adults (Dabul, 2000). The study received approval from the University of Queensland Medical Research Ethics Committee and the Queensland Health Human Research Ethics Committee.

### 2.2 Language assessment

Participants underwent language assessment at early subacute assessment (mean 27 days, range: 17–47 days). For each participant, spoken language comprehension (SpoComp) and spoken language production (SpoProd) performance were measured. The SpoComp score was derived from a combined score of the Auditory Word, Sentence, and Paragraph comprehension subtests from the Comprehensive Aphasia Test (CAT) (Swinburn et al., 2004). The SpoProd score was derived by combining the Fluency and Naming (nouns and verbs) CAT (Swinburn et al., 2004) subtests and a picture description task (Kertesz & Raven, 2007) (see Supplementary material, for details of the picture description task and analysis). Three individuals with aphasia were excluded from the SpoProd analysis as their picture description task was not administered at the early subacute stage. CAT (Swinburn et al., 2004) subtests (i.e., comprehension, fluency, naming) were double scored by experienced speech pathologists blinded to neurological and demographic data. Interrater reliability, available for speech production scores (fluency, naming) for one of the included studies, was 70% (calculated as a percentage of identical t scores).

### 2.3 Neuroimaging

#### 2.3.1 Neuroimaging protocol

Early subacute neuroimaging data were acquired between 2 and 6 weeks post‐stroke. Data from the first study (*N* = 13) (Roxbury et al., 2019) were collected using Siemens 3 Tesla Trio scanner (Siemens, Erlangen) with a 12‐channel head coil. During the same scanning session, a high‐resolution 3D T1‐weighted anatomical image [MP‐RAGE; TR 1900 ms; TE 2.4 ms; TI 900 ms; (0.9 mm)^3^ resolution] and 2D T2‐weighted FLAIR image (TE 87 ms, TR 9000 ms, TI 2500 ms, 36 3 mm slices, 0.9 × 0.9 mm in‐plane resolution) were acquired for each subject. Data from the second study (*N* = 24) were collected using a Siemens 3 Tesla MAGNETOM Prisma scanner (Siemens, Erlangen) using a 20‐channel head coil. A high‐resolution 3D T1‐weighted anatomical image [MP2RAGE; Marques et al., 2010; TR 4000 ms; TE 2.91 ms; TI1 700 ms; TI2 2220 ms; FA1 6°; FA2 7°; (1 mm)^3^ resolution] and 3D T2‐weighted FLAIR image [TE 386 ms, TR 5000 ms, TI 1800 ms, (1 mm)^3^ resolution] were acquired for each subject. Neither inspection of neuroimaging data, nor radiologist' report documented cases of midline shift.

#### 2.3.2 Corrected stroke lesion volume and lesion load to cortical language ROIs

Stroke lesion masks were manually delineated on high‐resolution T1‐weighted sequences in patient space using MRIcron (https://www.nitrc.org/projects/mricron) by two authors (K.G. and V.V.) and verified by two senior authors (K.M. and S.B.), blinded to behavioural and demographic data. The stroke lesion volume was calculated in native space for subsequent analyses. T2‐weighted FLAIR images were used to verify lesion location, particularly for haemorrhagic stroke. Stroke lesion load was defined as the ratio of lesion volume to intracranial volume, assessed for each patient before normalization.

For region of interest (ROI) analyses, T1‐weighted scan sequences and lesion masks were normalized to MNI space using enantiomorphic normalization (Nachev et al., 2008) in Clinical Toolbox (2012, https://www.nitrc.org/projects/clinicaltbx/) within SPM (version 12, https://www.fil.ion.ucl.ac.uk/spm/) in Matlab (version 2017, https://www.mathworks.com/). Normalized lesion masks were manually revised (K.G. and V.V.) where necessary and once again verified by two authors (K.M. and S.B.). Disagreements during the manual drawing process were resolved through group discussion (K.G., V.V., S.B., and K.M.).

Cortical language ROI masks in MNI space were created in DSI studio software (https://dsi-studio.labsolver.org), using the automated anatomical labelling atlas 3 (Rolls et al., 2020). Given the limited number of participants, the number of cortical ROIs was restricted to the four most frequently identified cortical regions associated with aphasia; Broca's area (*pars triangularis* + *pars opercularis*), *insula*, superior temporal gyrus (STG) and a combined region of both angular gyrus (AG) and supramarginal gyrus (SMG). Finally, the lesion ‘load’, or proportion damaged of each cortical ROI, was calculated by inclusively masking each cortical ROI with the normalized stroke lesion, and dividing it by the total volume of the ROI.

#### 2.3.3 WMH volume and proportion WMH load to callosal ROIs

For WMH volume and WMH lesion load to critical callosal ROIs, T2‐weighted FLAIR sequences were resliced and co‐registered to T1‐weighted sequences and normalized applying the T1‐weighted transformation matrix using fourth degree B‐spline interpolation (see above for T1‐weighted normalization). Upon evaluation of the accuracy of normalization in the ventricular region, we determined that seven participants' imaging data was not accurately normalized in this region. In three patients with asymmetrical ventricles, re‐normalization that excluded the entire left hemisphere resulted in improved normalization. In four patients, a ‘younger brain – age profile’ was noted compared to the rest of the cohort, and so their data was re‐normalized using a standard MNI template derived from a younger cohort from Clinical toolbox (2012, https://www.nitrc.org/projects/clinicaltbx/). This adjustment resulted in a modest improvement in registration in two cases. Re‐normalization to this younger standard template was not sufficient to improve normalization in the other two cases. All statistical analyses were therefore repeated after excluding these two participants, with no changes in the overall results. We have included these sub analyses in the Supplementary material.

WMH lesions were manually delineated on normalized T2‐weighted FLAIR sequences (https://www.nitrc.org/projects/mricron) by three authors (V.V., F.W., S.B.) and verified and amended as required by a radiologist (L.Z.), blinded to demographic and behavioural information. WMH lesions were only traced on the RH given the challenges of accurately tracing in the left hemisphere due to the extension of stroke lesion and associated pathological processes into the ventricular area. WMH volume (i.e., volume of total WMH lesion mask) was calculated for subsequent analyses. See Supplementary material for detailed description of WMH lesion delineation.

Callosal ROI analyses employed masks created in DSI studio software (https://dsi-studio.labsolver.org), based on the HCP1065 atlas (Yeh et al., 2018). Three callosal ROIs were selected and revised to include only their RH portions, CC‐Fmin, CC‐Fmaj, and CC‐Body. Finally, the proportion of each callosal ROI affected by the WMH was calculated by finding the volume common to both the WMH lesion mask and the callosal ROI (inclusive masking), then dividing by the total volume of the callosal ROI.

### 2.4 Statistical analyses

Relationships between the demographic and imaging characteristics were explored using Spearman correlations. Given that two cohorts were included, Wilcoxon signed‐ranked test was used to test for any differences between the datasets.

First, in Step 1, we used two stepwise linear regressions with forward selection to test which neuroimaging variables explained performance across both dependent variables (SpoComp and SpoProd) (i.e., one regression analysis for each dependent variable). The forward selection process starts with an empty model and iteratively adds and removes variables that contribute the most to improving the model fit until no additional variables significantly enhance the fit. The neuroimaging variables, including stroke lesion variables (i.e., corrected stroke lesion volume, lesion load Insula, lesion load Brocas, lesion load AG + SMG, lesion load STG) and WMH lesion variables (i.e., WMH volume, lesion load Fmin, lesion load Fmaj, lesion load Body), served as independent variables in both regression analyses. Due to the skewed nature of both stroke and WMH neuroimaging variables, these were transformed using square root transformation. The neuroimaging variables that demonstrated significant explanatory power for the outcomes identified in Step 1 were retained for inclusion in Step 2.

In Step 2, we performed a standard multiple linear regression to test the relative importance of the significant neuroimaging variables (Step 1) and stroke‐related demographic variables (i.e., age, sex) to identify the best combination of predictors for the two language outcomes (SpoComp and SpoProd). Our analyses included two apriori steps (Steps 1 and 2) for two outcome measures (SpoComp and SpoProd), as such the alpha level of each model was set to *p* = .012 (0.05/4, Bonferroni‐correction).

The assumptions of linearity of the data, normality of residuals, independence of residuals and homoscedasticity were all met (see Supplementary material for diagnostic tests and plots). All analyses were conducted in IBM SPSS Statistics for Windows, Version 22.0.

## 3 RESULTS

**FIGURE 1:**
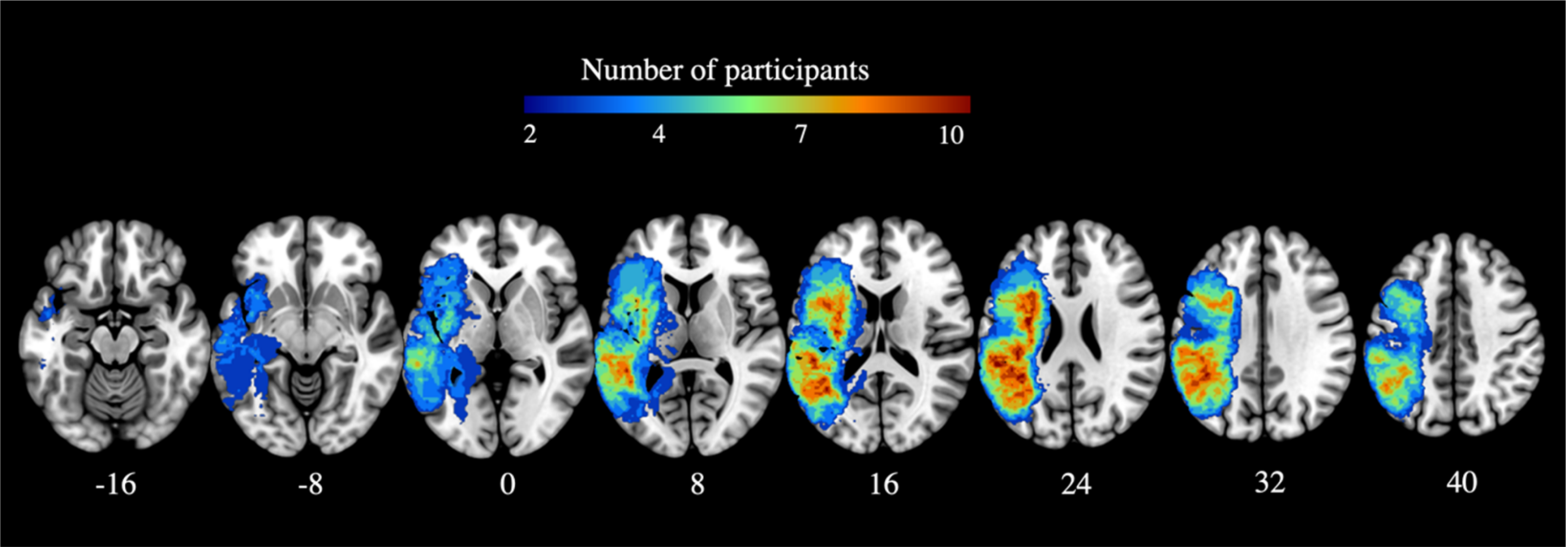
Stroke lesion overlay at the early subacute stage (*N* = 37). Warmer colours indicate greater areas of lesion overlap. Z plane coordinates (mm) are reported in MNI space.

**FIGURE 2:**
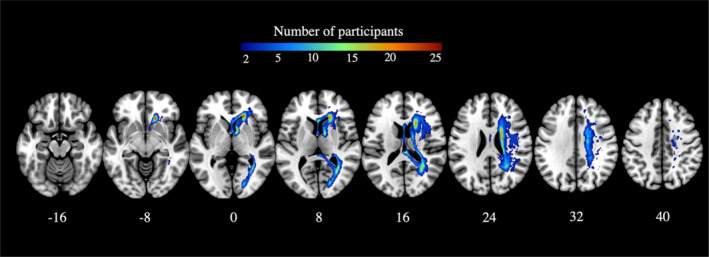
WMH lesion overlay at the early subacute stage (*N* = 37). Warmer colours indicate greater areas of lesion overlap. Z plane coordinates (mm) are reported in MNI space.

**FIGURE 3:**
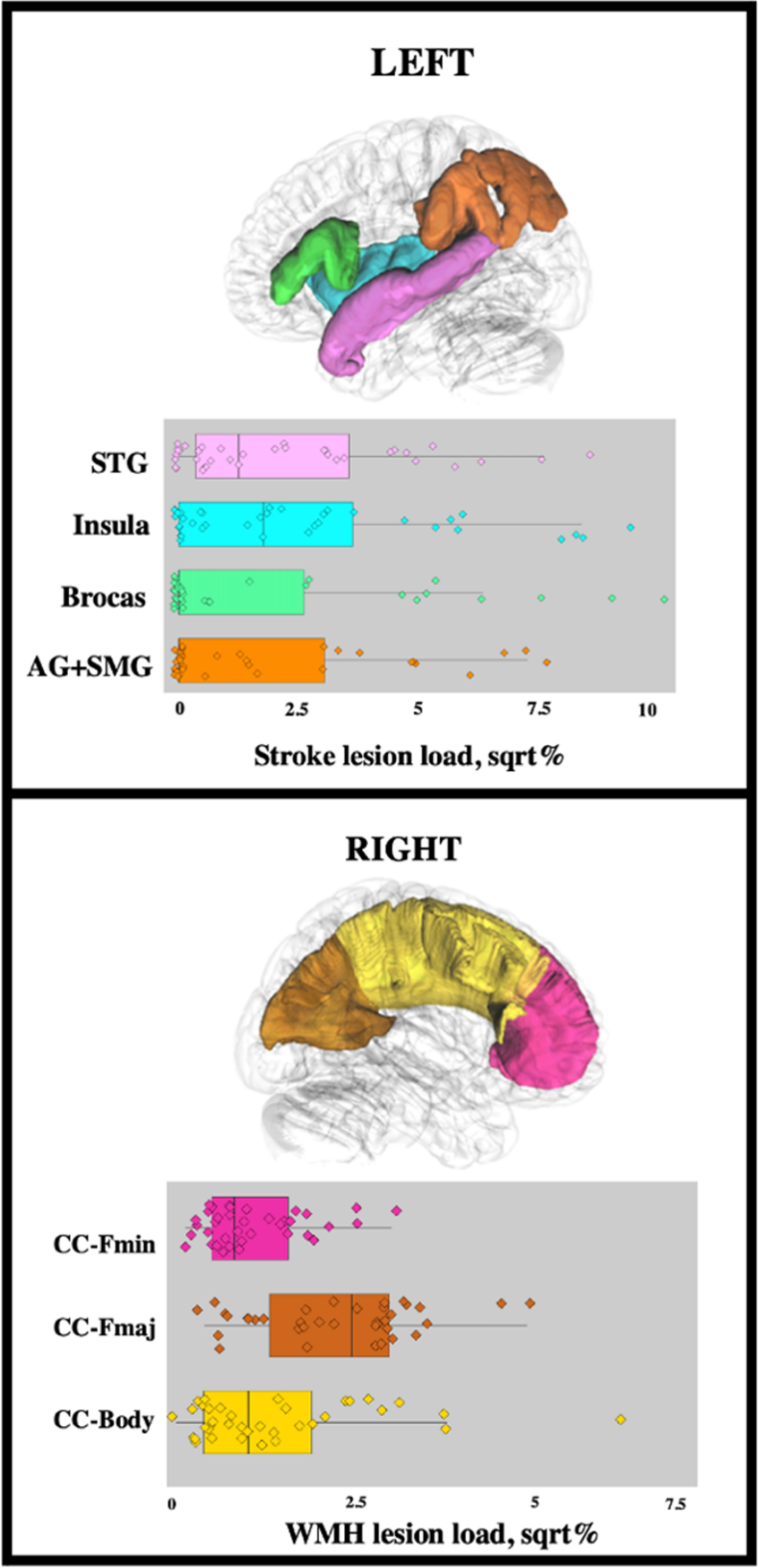
Transformed stroke lesion load across cortical ROIs and transformed WMH lesion load across callosal ROIs. AG + SMG, angular gyrus + supramarginal gyrus; CC‐Body, corpus callosum body; CC‐Fmaj, corpus callosum forceps major; CC‐Fmin, corpus callosum forceps minor; sqrt, square root; STG, superior temporal gyrus; WMH, white matter hyperintensities.

All demographic, stroke lesion, and WMH variables and outcomes can be found in Table 1.^1^ Overlay maps of all stroke lesions can be found in Figure 1, and WMH lesions can be found in Figure 2. Corrected stroke lesion volume did not correlate with WMH volume (*p* = .762) or any of the callosal ROIs (CC‐Fmin: *p* = .482, CC‐Fmaj: *p* = .971, CC Body: *p* = .997). WMH lesion load within callosal ROIs strongly correlated with total WMH volume (CC‐Fmin: *r* = .88, CC F‐maj: *r* = .82, CC‐Body = .93). Figure 3 illustrates the distribution of transformed stroke lesion loads and transformed WMH lesion loads in relevant ROIs.

**TABLE 1:** Characteristics: Overview of socio‐demographic variables, medians (range) of neuroimaging variables and outcomes in the whole sample and between dataset comparisons.

Characteristic	Whole sample median (range)	Dataset 1 median (range)	Dataset 2 median (range)	Wilcoxon signed‐rank test
	*N* = 37	*N* = 24	*N* = 13	(Between datasets)
Sex, female/male	10/17	6/18	4/9	.72
Stroke type, isch./hem.	29/8	19/5	10/3	.89
Age, y	66 (42–86)	65.5 (42–86)	66 (51–84)	.97
Education level^a^	4 (0–4)	4 (0–4)	2 (0–4)	.03
SLC score, early subacute	54 (32–66)	53.50 (36–66)	55 (32–65)	.28
SLP score, early subacute	32.25 (0–66.07)	28.31 (6–64.52)	37. 22 (0–66.07)	.26
Stroke lesion load AG + SMG, sqrt	0 (0–7.51)	0 (0–7.48)	.78 (0–3.24)	.74
Stroke lesion load Broca's area, sqrt	0 (0–9.71)	0 (0–9.71)	0 (0–7.24)	.52
Stroke lesion load insula, sqrt	1.71 (0–9.06)	1.73 (0–9.06)	1.71 (0–7.79)	.41
Stroke lesion load STG, sqrt	1.21 (0–8.28)	1.79 (0–8.28)	.57 (0–4.45)	.14
Corrected stroke lesion volume, sqrt	1.13 (.04–2.78)	1.22 (.20–2.78)	.96 (1.63–4.71)	.22
WMH lesion load CC‐Fmin, sqrt	.78 (.12–2.90)	.75 (.12–2.06)	.89 (.31–2.90)	.16
WMH lesion load CC‐Fmaj, sqrt	2.36 (.51–4.71)	1.74 (.51–3.36)	2.76 (1.63–4.71)	.01
WMH lesion load CC‐Body, sqrt	.97 (0–5.95)	.64 (0–3.64)	1.19 (.28–5.95)	.13
WMH lesion volume, sqrt cm^3^	2.58 (.73–8.10)	2.37 (.73–4.53)	2.90 (1.92–8.10)	.02
*Raw medians of neuroimaging variables*				
Stroke lesion load AG + SMG	0 (0–56.48)	0 (0–56.48)	.61 (0–19.86)	—
Stroke lesion load Broca's area	0 (0–94.38)	0 (0–94.38)	0 0–52.51)	—
Stroke lesion load Insula	2.95 (0–82.17)	3.10 (0–82.17)	2.95 (0–60.78)	—
Stroke lesion load STG	1.48 (0–68.58)	3.40 (0–68.58)	.33 (0–19.86)	—
Corrected stroke lesion volume	1.23 (0–7.75)	1.50 (0–7.75)	.92 (2.68–22.26)	—
WMH lesion load CC‐Fmin	.61 (.01–8.41)	.56 (.01–4.25)	.79 (.10–8.41)	—
WMH lesion load CC‐Fmaj	5.58 (0.27–22.26)	3.04 (.27–11.31)	7.76 (2.68–22.26)	—
WMH lesion load CC‐Body	.94 (0–35.44)	.43 (0–13.30)	1.42 (.08–35.44)	—
WMH lesion volume, cm^3^	6.70 (.54–65.77)	5.65 (.52–20.55)	8.43 (3.71–65.77)	—

Abbreviations: AG + SMG, angular gyrus + supramarginal gyrus; CC‐Body, corpus callosum body; CC‐Fmaj, corpus callosum forceps major; CC‐Fmin, corpus callosum forceps minor; hem, haemorrhagic; isch, ischaemic; SLC, spoken language comprehension; SLP, spoken language production; sqrt, square root; STG, superior temporal gyrus; WMH, white matter hyperintensities; y, years.

^a^ Educational levels scored according to the UNESCO ISCED (International Standard Classification of education) classification system (Statistics, 2011).

Approximately 20% of patients experienced a haemorrhagic stroke. Univariate linear regression analysis indicated that there was a difference in SpoComp scores between ischaemic and haemorrhagic stroke subgroups (*p* = .01), but not SpoProd scores (*p* = .08). As such, stroke type was included as a predictor in analyses as a co‐variate (Step 2), together with age and sex.

Given the two cohorts had different imaging parameters, we investigated for any significant differences between behavioural or neuroimaging measures. Non‐parametric Wilcoxon signed‐rank test was used to assess between population differences due to skewed distribution. Significant between‐dataset differences were observed for Education, WMH lesion volume and WMH lesion load CC‐Fmaj (see Table 1).

### 3.1 SpoComp score

First, we conducted a stepwise multiple linear regression with forward selection to determine which neuroimaging variables accounted for the variance in SpoComp scores. The corrected stroke lesion volume and WMH lesion load within CC‐Fmin were significant predictors of the SpoComp score, together accounting for 40% variance in SpoComp scores (see Table 2).^2^ No other neuroimaging variables explained the variance (see Supplementary material for detailed results of Step 1).

**TABLE 2:** Results of significant neuroimaging variables from the stepwise regression (Step 1) for SpoComp and SpoProd scores.

	Model	Variable	*F*_(df)_	*p*	*R* ^2^	*R* ^2^ change
SpoComp	1	Corrected stroke lesion load	*F*_(1,35)_ = 8921	.005	.203	.203
	2	WMH lesion load CC‐Fmin	*F*_(1,34)_ = 11,447	<.001	.402	.199
SpoProd	1	Corrected stroke lesion load	*F*_(1,32)_ = 9109	.005	.222	.222

Abbreviations: CC‐Fmin, corpus callosum forceps minor; SpoComp, spoken comprehension; SpoProd, spoken production; WMH, white matter hyperintensities.

Next (Step 2), we conducted a standard regression analysis that included the significant neuroimaging variables (i.e., corrected stroke lesion volume, WMH CC‐Fmin) as well as demographic variables (i.e., age, sex, stroke type). This approach was conducted to test the relative importance of individual variables of interest associated with SpoComp scores (see Table 3 for statistics). Here, corrected stroke lesion volume, stroke type and WMH lesion load within CC‐Fmin significantly predicted SpoComp scores (model statistics: *R* ^2^ = .66, *F*(5, 31) = 11.98, *p* < .001). Patients with an ischaemic stroke had, on average, 9.78 lower early subacute SpoComp scores than patients with haemorrhagic stroke (*t* = −3.64, *p* < .001). The model further indicated that higher WMH lesion load in CC‐Fmin was associated with a decrease in SpoComp scores (*β* = −5.37, *t* = −2.59, *p* = .01). Finally, a higher corrected stroke lesion load was associated with a decrease in SpoComp score (*β* = −8.30, *t* = −4.50, *p* < .001). Age and sex did not independently explain variance in outcomes.

**TABLE 3:** Individual variables used in the multiple regression analysis (Step 2), including significant neuroimaging variables (Step 1) and socio‐demographic variables.

	*β*	St error	*t*	*p*
*Dependent variable*: *SpoComp score R* ^2^ = .*66*, *F*(*5*, *31*) = *11*.*98*, *p* < .*001*				
Intercept	74.76	8.08	9.25	**<.001**
Age	−.22	0.12	−1.71	.09
Stroke type, isch.	**−9.78**	**2.68**	**3.64**	**<.001**
Sex, f	3.61	2.42	1.48	.14
WMH CC‐Fmin, sqrt	**−5.37**	**2.07**	**−2.59**	**.01**
Corrected stroke lesion volume, sqrt	**−8.30**	**1.84**	**−4.50**	**<.001**
*Dependent variable*: *SpoProd score R* ^2^ = .*38*, *F*(*4*, *29*) = *4*.*60*, *p* = .*005*				
Intercept	74.25	19.66	3.77	**<.001**
Age	−.51	.25	−1.98	.05
Stroke type, isch.	10.44	7.01	1.48	.14
Sex, f	8.33	6.27	1.32	.19
Corrected stroke lesion volume, sqrt	**−16.57**	**−4.83**	**−3.42**	**<.01**

*Note*: Bold values denote variables that significantly explain variance in outcomes.

Abbreviations: f, female; Isch, ischaemic; SpoComp, spoken comprehension; SpoProd, spoken production; sqrt, square root; WMH CC Fmin, white matter hyperintensities within corpus callosum forceps minor.

### 3.2 SpoProd score

To determine which neuroimaging variables accounted for the variance in SpoProd scores, we conducted a stepwise linear regression with forward selection with all neuroimaging variables. Only corrected stroke lesion volume emerged as a significant predictor of the SpoProd score, accounting for only 22% variance in SpoProd scores (see Table 2 for statistics). No other neuroimaging variables explained the variance (see Supplementary material for detailed results of Step 1).

Next, we conducted a standard regression analysis that included the significant neuroimaging variables (i.e., corrected stroke lesion volume) as well as demographic variables (i.e., age, sex, stroke type) to test their relative importance associated with SpoProd scores. Here, only stroke lesion volume significantly predicted SpoProd scores (model statistics: *R* ^2^ = .38, *F*(4, 29) = 4.60, *p* = .005) accounting for a considerable proportion of the variability in SpoProd scores. A higher corrected stroke lesion load was associated with a decrease in SpoProd score (*β* = −16.57, *t* = −3.42, *p* = .001). Age, sex and stroke type did not explain variance in outcomes.

## 4 DISCUSSION

In this study, we investigated the impact of early subacute WMH metrics, a surrogate of premorbid volume and distribution, on the inter‐individual variability observed in post‐stroke aphasia outcomes. We probed the contribution of the total volume of WMH within the contralesional RH and the WMH lesion load within an empirically motivated tract, the corpus callosum.

We illustrate, for the first time, that premorbid WMH distribution negatively impacts early subacute aphasia outcomes after stroke. A key finding of this research is the association between premorbid WMH load within the CC‐Fmin and early subacute comprehension impairments, when considered with other stroke lesion and demographic variables. This negative impact of callosal WMH on language in aphasia is consistent with converging evidence from ageing (Camerino et al., 2020; Freeze et al., 2022; Vemuri et al., 2021) and other stroke populations (Zhang et al., 2014) suggesting that WMH disrupts neural networks that underpin a range of cognitive functions, resulting in behavioural consequences. We found no relationship between the total volume of WMH and aphasia outcomes. Our results indicate that rather than total WMH volume, localization of WMH burden, that is, the extent of the damage within the CC‐Fmin, may be a more sensitive biomarker of structural brain health.

Furthermore, we provide novel evidence that WMH anatomical distribution affects language skills differently, with the WMH lesion load within CC‐Fmin negatively impacting language comprehension, but not language production. This increased vulnerability of spoken comprehension to WMH and other brain ageing markers  highlights that the different domains of language need to be examined separately in order to derive clinically meaningful and sensitive neurobiological biomarkers of language recovery after stroke (Wilson et al., 2023).

Finally, our results indicated that individuals that had an ischaemic stroke had higher subacute SpoComp scores than those that had a haemorrhagic stroke. Although somewhat underpowered, this observation likely reflects the nature of the stroke and differences in lesion neuroanatomy between the two groups. Furthermore, we found no evidence of an association between stroke type and subacute SpoProd scores.

### 4.1 Impact of WMH within CC‐Fmin

In research on cognitive ageing, the distribution of WMH within several segments of the CC, particularly the CC‐Fmin and the CC‐Fmaj, has been shown to independently explain the cognitive sequelae of WMH pathology, suggesting that callosal WMH may act as a surrogate marker of cognitive ageing processes (Freeze et al., 2022; Petersen et al., 2022; Vemuri et al., 2021). Our findings indicate that this may also be the case in pathological populations, such as post‐stroke aphasia.

The CC‐Fmin traverses and connects several regions that lie within the bilateral prefrontal cortices (PFCs), including the orbitofrontal, cingulate, and the superior frontal cortices. The contribution of the frontal white matter in healthy and pathological ageing has been a topic of extensive research (Brickman et al., 2012; Fjell et al., 2017; Schneider et al., 2022). Recent analyses of microstructural indices in large cohort samples confirmed that frontal white matter is singularly vulnerable to microstructural changes as we age and that these changes are predictive of worse cognitive performance and further cognitive decline (Poulakis et al., 2021; Saboo et al., 2022; Vemuri et al., 2021). From a network perspective, segments of the frontal white matter may constitute ‘key hubs’ (Stam, 2014) or ‘bottlenecks’ (Griffis et al., 2017), that is, brain structures that are preferentially afflicted across disorders, with damage to them being disproportional associated with psycho‐neurological disturbance (van den Heuvel & Sporns, 2019). It is worth noting that the median percentage WMH lesion load within CC‐Fmin was 0.61% (range 0.01–8.41%) (see Table 1), indicating that the distribution of WMH lesions within frontal callosal connections is generally modest, with only a small sub‐set of patients exhibiting more pronounced lesion within the tract. Coupled with the absence of an identified relationship between WMH lesion load and other CC segments, this finding suggests that frontal callosal connections may play a strategic role in cognitive networks, with minor disruptions resulting in behavioural consequences.

In the case of post‐stroke aphasia, the exact neurobiological mechanisms by which callosal WMH burden predisposes individuals with aphasia to suboptimal recovery from aphasia remains unclear. The functional roles of the CC segments in language recovery remain to be determined. CC is seldom affected by ischaemia given its rich blood supply from various arteries (Chrysikopoulos et al., 1997) and aphasia research has almost exclusively focused on delineating the intrahemispheric white matter of the language network, identifying tracts such as the superior longitudinal fasciculus and the inferior frontal longitudinal fasciculus as vital to successful language recovery after stroke (Ivanova et al., 2016; Zhang et al., 2021). Conversely, more recent studies that have used whole‐brain analyses identified associations between CC microstructure, unaffected by the stroke injury directly, and language outcomes after stroke (Dresang et al., 2021; Hula et al., 2020; Pani et al., 2016), further highlighting the need to consider how the structural integrity of the CC contributes to aphasia recovery.

Regions within the PFC have been robustly implicated in many large‐scale neural networks such as the fronto‐parietal network, the salience network, the cingulo‐opercular network, and the default mode network (for a review, see Menon & D'Esposito, 2022). Several of these neural networks have, in turn, been associated with language abilities in aphasia (Brownsett et al., 2014; Geranmayeh et al., 2016; Geranmayeh et al., 2017). Our findings suggest that callosal WMH may act to influence compensatory processes, or the upregulation of neural networks supporting the recovery of language. If language abilities in aphasia are, in part, contingent on effective domain‐general compensatory or upregulatory processes reliant on spared interhemispheric connections, the disruption that may occur with the presence of WMH lesions within these connections may contribute to suboptimal recovery of language.

### 4.2 Lack of impact of total WMH volume

In clinical practice, WMH were first discerned as regions of diffuse hyperintense signal on T2‐weighted FLAIR images and therefore assessed using qualitative clinical severity scales (Fazekas et al., 1987; Scheltens et al., 1993). Despite the robust evidence that qualitative WMH scales capture clinically pertinent differences in WMH burden (for reviews and meta‐analyses, see Georgakis et al., 2019; Hamilton et al., 2021), recent research highlights the advantage of precise and systematic volumetric analyses in quantifying WMH burden and its relationship with cognition in ageing (Hawe et al., 2018; Kaskikallio et al., 2019). The role of WMH volume in populations with other primary neurological disorders, such as dementia or post‐stroke aphasia, is less well characterised. In line with a handful of studies investigating other domains of cognition after stroke (Ferris et al., 2022; Röhrig et al., 2022), we failed to observe an association between premorbid WMH volume and stroke outcomes. Conversely, several larger studies reported significant associations between WMH volume and cognition after stroke (Clancy et al., 2022; Hawe et al., 2018). These inconsistent findings may partially reflect the well‐accepted challenge of extreme heterogeneity in clinical stroke cohorts which can conceal significant predictors of impairment and recovery, especially when complex and partially multi‐collinear relationships exist between predictors (Boyd et al., 2017). Furthermore, a recent large study by Bonkhoff et al. (2022) (*n* > 1100) showed that severe WMH predisposed individuals with stroke to worse acute functional outcomes only if the stroke lesions were located in a subset of specific brain territories, which included frontal language network regions, suggesting a complex interaction of stroke and WMH variables. Combined with our results, these findings challenge the assumption that the stroke lesion volume and the WMH lesion volumes contribute to the observed behavioural impairment in a simple linear and additive fashion for all individuals with aphasia across all timepoints. Future studies must endeavour to minimize variance in patient cohorts, by considering not only the volume of the stroke lesion but also its location, and thus increase the power to detect sensitive biomarkers of recovery (Boyd et al., 2017).

### 4.3 Increased vulnerability of spoken comprehension to WMH

The unique effect that WMH may have on the impairment and the recovery of different language skills is an exciting avenue for future research. In line with previous studies (Basilakos et al., 2019; Varkanitsa et al., 2020), this study found no effect of WMH on language production skills. Conversely, Wright et al. (2018) identified an association between naming and fluency and WMH burden. These discordant results are not restricted to aphasia research, with similar discrepancies within other cognitive domains, such as memory or attention (Liang et al., 2019; Nakamori et al., 2020). These inconsistencies could partially reflect the range of challenges faced by the field including; insufficient statistical power to detect the effect of WMH burden, and large methodological variation across studies (e.g., differences in stroke lesion distribution and severity, different behavioural language measures, different covariates), but could also be partially explained by emerging evidence indicating that some individual cognitive domains may be more vulnerable to WMH burden than others (Hamilton et al., 2021).

When compared to production skills, longitudinal changes in spoken comprehension was identified as more vulnerable to the effects of WMH pathology and other brain ageing markers in a previous study (Kristinsson et al., 2022). The contribution of executive processing to sentence level comprehension tasks has been shown in behavioural studies with healthy participants (Caplan et al., 2013; Gajardo‐Vidal et al., 2018; Key‐DeLyria Sarah & Altmann Lori, 2016; Yoon et al., 2015). From an anatomical perspective, some regions within the bilateral PFC have been implicated in both sentence level comprehension and executive processing tasks in both healthy participants (Gajardo‐Vidal et al., 2018; Key‐DeLyria & Altmann, 2016; Seeley et al., 2007; Walenski et al., 2019) and people with aphasia (Brownsett et al., 2014; Stefaniak et al., 2021; van Oers et al., 2010). Given the evidence demonstrating that WMH lesions compromise connections that project to the PFC, any functional relationship between language comprehension and executive processing is likely to be more susceptible to the cumulative effects of premorbid WMH burden.

### 4.4 Limitations

While we present a well‐controlled investigation of the relationship between both WMH volume and its distribution and post‐stroke aphasia, our findings are limited by the sample size and the merging of two different datasets. This may have impacted on the identification of additional associations between volumetric WMH variables and language measures.

Post‐stroke aphasia represents a network disorder arising from injury within multiple cortical, white matter, and subcortical structures (Thiel & Zumbansen, 2016). Conducting an extensive analysis involving numerous language ROIs falls beyond the scope and statistical feasibility of the present investigation. Specific lesion topography within the language network has been shown to serve as a predictor of different language impairments in aphasia (Crinion et al., 2013; Fridriksson et al., 2018; Wilson et al., 2023). Consequently, larger‐scale investigations are required to establish whether more nuanced assessment of primary stroke lesion (i.e., assessment of a higher number of cortical and subcortical language network ROIs) can refine our understanding of the impact of premorbid WMH.

Second, WMH are a radiological manifestation of global white matter disease that simultaneously affects multiple white matter connections (ter Telgte et al., 2018). WMH damage restricted to a single tract is rarely observed and as such it is challenging to assign functional roles to specific WMH affected tracts. However, despite the diffuse nature of WMH, WMH specifically within the CC‐Fmin have been frequently associated with cognitive decline in pathological ageing (Biesbroek et al., 2016; Biesbroek et al., 2020; Camerino et al., 2020; Duering et al., 2011; Duering et al., 2014; Freeze et al., 2022; Hilal et al., 2021; Jiang et al., 2018; Lampe et al., 2019; Vemuri et al., 2021; Zhao et al., 2018), making frontal callosal connections an ideal target for future investigations.

This study included two datasets with distinct neuroimaging parameters which undeniably introduces noise into the derived metrics and subsequent analyses. However, it is important to consider this limitation within the wider context of the substantial benefits that can be gained from the combination of datasets to create adequately large groups for the identification of potential imaging biomarkers. Individual research groups face tremendous challenges in acquiring sufficiently large datasets within heterogeneous phenotype groups. Most aphasia cohorts, particularly those with early (acute, subacute) behavioural and neuroimaging timepoints, have had limited cohort sizes, seldom surpassing 25–30 participants (Stefaniak et al., 2022; Stockert et al., 2020). The concept of combining datasets has emerged as a potential research direction to reduce research waste and harness the availability of smaller existing datasets (Hayward et al., 2022). Future single or multi‐research centre studies with identical neuroimaging parameters are required to confirm and likely refine our findings.

Finally, our study comprised a patient cohort with a notable chronological age range (range 42–86 years), which exhibited distinct ‘brain‐age profiles’. The ventricular region is susceptible to substantial inter‐individual variations in ageing which can lead to challenges in accurate normalization. As a result, the application of standard template ROIs, such as the callosal ROIs used in this study, some overestimation and underestimation of true WMH burden is unavoidable. There is no consensus on the inclusion of the midline section when considering WMH (Duering et al., 2011; Röhrig et al., 2022). We decided to include the midline section within our analysis due to our specific interest in the WMH lesion distribution, particularly within the corpus callosum.

### 4.5 Future research

Research into the role of white matter health in aphasia outcomes is in its infancy and further research is needed to (a) improve the sensitivity and reliability of WMH as biomarkers of stroke outcomes, and (b) gain insights into the mechanisms by which the recovery processes may be disrupted by the presence and the severity of WMH lesions.

From a biomarker perspective, the ubiquity of WMH in older age suggests that WMH are not exclusively pathological (Raja et al., 2019), so it is essential to identify quantitative criteria that can more reliably differentiate healthy and pathological WMH burden and distribution in the context of disruption to function in combination with stroke injury.

The disproportionate involvement of the CC‐Fmin across white matter health research (Biesbroek et al., 2016; Biesbroek et al., 2020; Camerino et al., 2020; Duering et al., 2011; Duering et al., 2014; Hilal et al., 2021; Jiang et al., 2018; Lampe et al., 2019; Zhao et al., 2018) suggests that WMH within frontal callosal connections may constitute a reliable cross‐diagnostic proxy of pathological WMH profiles. Our study did not consider long association fibres of the bilateral left‐asymmetric language network (Forkel & Catani, 2019), because these tracts are less frequently affected by radiographic WMH lesions (Biesbroek et al., 2017). Our study, along with previous research in pathological aging (Camerino et al., 2020), revealed significant associations between language comprehension and verbal‐executive skills and callosal WMH lesions. We suggest that this demonstrates that pathology outside the core language network can contribute to language dysfunction in aging and in stroke. However, we cannot exclude the possible contributions to outcome of WMH lesions within the language‐network itself and future research should specifically investigate the distribution of WMH lesion loads within both callosal connections and strategic language‐network association fibres.

Given that WMH were identified as localized structural lesions, the exact neurobiological mechanisms by which WMH burden predisposes individuals with aphasia and other stroke survivors (Georgakis et al., 2019) to less favourable recovery remains unknown.

Conceptually, WMH load may be inferred to reflect reduced structural brain health which likely weakens optimal recovery processes (Kristinsson et al., 2022; Umarova, 2017). However, this has not been empirically corroborated and we are not aware of any study that has complemented structural WMH data with functional network engagement in stroke recovery research to target this hypothesis more explicitly. It has been proposed that bilateral domain general compensation may underpin language recovery (Brownsett et al., 2014; Geranmayeh et al., 2017; Schneider et al., 2022) and future research can feasibly investigate whether the supportive role of domain‐general neural networks is modulated by total or tract‐specific WMH burden.

Finally, a crucial aspect that warrants additional investigation is the potentially unique relationship between spoken comprehension and ageing neuroimaging biomarker, such as WMH burden. In a seminal lesion‐symptom study, encompassing the largest cohort of individuals with aphasia to data (*n* > 200), age was identified to contribute to comprehension outcomes in aphasia more than any other language skill, albeit with a modest effect (Wilson et al., 2023). Given that age is consistently linked to neuroimaging markers of aging, including WMH burden (Prins & Scheltens, 2015), these findings further suggest that language comprehension may be particularly susceptible to the effects of aging (Wilson et al., 2023).

## 5 CONCLUSION

This study builds on previous findings reliant on qualitative assessments of WMH burden by presenting the first investigation of the relationship between quantitative measures of early subacute WMH, a surrogate of premorbid levels, and comprehensive measures of language in post‐stroke aphasia. We extend the robustly replicated finding that callosal WMH plays a critical role in pathologically ageing groups (Biesbroek et al., 2016; Biesbroek et al., 2020; Camerino et al., 2020; Duering et al., 2011; Duering et al., 2014; Hilal et al., 2021; Jiang et al., 2018; Lampe et al., 2019; Zhao et al., 2018) and confirm that measures of frontal callosal WMH volume reliably improves explaining variability of outcomes across another pathological group, namely post‐stroke aphasia. While WMH topography is rarely considered in stroke (Röhrig et al., 2022) and the assessment of the entire extent of WMH pathology is the most prevalent WMH measure (Basilakos et al., 2019; Varkanitsa et al., 2020; Wright et al., 2018), our findings argue in favour of tract‐specific WMH lesion load indices in explaining variance in outcomes in post‐stroke aphasia.

From a clinical perspective, frontal callosal WMH may constitute a vital cross‐diagnostic imaging biomarker of reduced structural brain health and therefore index suboptimal recovery of language after stroke. The inclusion of callosal WMH, along with additional neuroimaging biomarkers that impact aphasia recovery, may contribute to more reliable predictions, and therefore the provision of more meaningful prognoses. Future large‐scale studies are required to confirm the predictive role of frontal callosal WMH in the recovery of language comprehension, and the differential susceptibility of some language skills over others.

## FUNDING INFORMATION

Financial support for the work was provided by the National Health and Medical Research Council (#1104194), NHMRC‐funded Centre of Research Excellence in Aphasia Recovery and Rehabilitation (#1153236), Finnish Cultural Foundation (#191230), Maire Taponen Foundation, Orion Research Foundation sr, and Signe and Ane Gyllenberg Foundation.

## CONFLICT OF INTEREST STATEMENT

The authors declare that there is no conflict of interest.

## Supporting information


**Data S1:** Supporting Information.

## Data Availability

The data that support the findings of this study are available on request from the corresponding author. The data are not publicly available due to privacy or ethical restrictions.

## Footnotes

^1^
The UNESCO ISCED classification system (Statistics, 2011) is scored as follows 0: early childhood, 1: primary education, 2: lower secondary education, 3: upper secondary education, 4: post‐secondary non‐tertiary education, 5: short‐cycle tertiary education, 6: bachelor's or equivalent, 7: master's or equivalent, 8: doctoral or equivalent.

^2^
Used to determine which neuroimaging variables explained variance in outcomes.
