# Supplementary material for: The volume and the distribution of premorbid white matter hyperintensities: Impact on post‐stroke aphasia
Source: Hum Brain Mapp. 2024 Jan 15;45(1):e26568. doi: 10.1002/hbm.26568 (PMC10789210; doi:10.1002/hbm.26568)

SUPPLEMENTARY MATERIAL

**The volume and the distribution of premorbid white matter hyperintensities: impact on post-stroke aphasia**

Vadinova V.^1,2,3^, Sihvonen A.J.^1,2,3,4,5^, Wee, F.^1^, Garden K.L.^1,2,3^, Ziraldo, L.^1^, Roxbury T.^1^, O’Brien K.^1^, Copland D.^1,2,3^, McMahon K.L.^6^, Brownsett S.L.E.^1,2,3^

1 Queensland Aphasia Research Centre, University of Queensland, Australia

2 School of Health and Rehabilitation Sciences, University of Queensland, Australia

3 Centre of Research Excellence in Aphasia Recovery and Rehabilitation, La Trobe

University, Australia

4 Cognitive Brain Research Unit (CBRU), University of Helsinki, Finland

5 Centre of Excellence in Music, Mind, Body and Brain, University of Helsinki, Finland

6 School of Clinical Sciences, Centre for Biomedical Technologies, Queensland

University of Technology, Australia

**Corresponding author:**

Veronika Vadinova

Queensland Aphasia Research Centre, University of Queensland, Australia

St Lucia 4072

Brisbane, Australia

v.vadinova@uqconnect.edu.au

+61 0490916670

**1. Calculation of Spoken Production score.**

*Fluency, Naming assessments*

All raw scores from the Comprehensive Aphasia Test (CAT) subtests were independently scored by two qualified speech pathologists blinded to neurological data.

*Picture description*

Transcriptions of picture descriptions from the Western Aphasia Battery (WAB) (ref) were coded by two researchers blinded to neurological, demographic and timepoint data according to the procedures in the Codes for the Human Analysis of Transcripts (CHAT) (<https://talkbank.org/>). These coded transcriptions were then analysed using the Computerized Language Analysis (CLAN) (https://www.talkbank.org/) software, which enables automatic computation of various linguistic indices. We derived three indices to capture the lexical (informativeness), grammatical (*grammatical wellformedness)* and fluency (speed) properties of language produced using this stimulus*.* Speed (number of intelligible words per minute) and grammatical wellformedness (percentage of grammatically correct utterances produced, excluding empty speech, jargon and irrelevant content) were automatically calculated by the CLAN software. Informativeness was assessed by calculating the number of information carrying units (ICU). ICU's were identified separately on uncoded transcripts by two researchers blinded to neurological data, including a speech pathologist. Any disagreements in ICU identified were resolved by consensus. An informativeness index was calculated as the number of accurate ICU's minus all inappropriate ICUs (such as; phonological paraphasias, semantic paraphasias, neologisms, circumlocutions) per minute. Final SpoProd measure was calculated as follows:

$SpoProd score = \frac{fluency + naming + speed + grammatical wellformedness + informativeness}{5}$

**2. WMH lesion delineation**

WMH lesions were delineated on normalized 3D T2-weighted FLAIR images using axial slices based on the hyperintensity compared to normal WM. Coronal and sagittal slices were consulted to determine the extent of damage. As WMH lesions predominantly occur in the periventricular watershed area, with often noticeable protrusion around the lateral ventricle, caution was taken to differentiate between striatal areas and WMH lesions alongside the lateral ventricle. All delineated WMH lesions were confirmed by a radiologist. For 19 participants, minor corrections (i.e. overestimation by 1-2 voxels, incidental inclusion of head of the caudate in the lesion mask) were recommended and applied under their guidance.

**3. Stroke lesion delineation**

Stroke lesions were defined by comparison of intensity with the homologous region, consulting both T1-weighted and T2-weighted FLAIR images. This encompassed necrotic tissue (iso intense with CSF on T1- weighted images), peri-necrotic (hypointense on T1 - weighted images), the presence of blood in haemorrhagic stroke (hyperintense on T1- weighted images) and perilesional hyperintensities on T2-weighted FLAIR images. Two researchers (VV and KG) manually drew these lesions, which were then confirmed by researchers blinded to behavioural and demographic data and experienced in lesion delineation (KM, SB). For 21 participants, minor corrections (e.g., reducing lesion mask edges by 1-2 voxels) were carried out by these researchers through group consensus.

**4. Analyses excluding patient 13 and patient 40**

Model statistics: early subacute SpoComp score: R2 = .69, F(5, 29) = 13.22, p < .001

Model statistics: chronic SpoComp score: R2 = .36, F(4, 27) = 3.28, p = .013

**Table 1**

Individual predictors of final Model 2 that included significant neuroimaging variables and socio-demographic variables after exclusion if two participants.

| **SpoComp score**  ***(model statistics: R^2^ = .69, F(5, 29) = 13.22, p < .001)*** | | | | |
| --- | --- | --- | --- | --- |
|  | ***β*** | **St Error** | ***t*** | ***p*** |
| Age | -0.16 | .11 | -1.34 | .18 |
| Stroke type, isch. | **-9.44** | **2.44** | **3.86** | **< .001***** |
| Sex, fem | 2.65 | 2.23 | 1.18 | .24 |
| CC Fmin | **-6.36** | **1.90** | **-3.32** | **< .01*** |
| Corrected stroke lesion volume | **-8.30** | **1.84** | **-4.50** | **< .001***** |
|  | | | | |
| **SpoProd score**  ***(model statistics: R^2^ = .36, F(4, 27) = 3.28, p = .013)*** | | | | |
|  | ***β*** | **St Error** | ***t*** | ***p*** |
| Age | -0.50 | .26 | -1.91 | .06 |
| Stroke type, isch. | 9.94 | 7.20 | 1.37 | .17 |
| Sex, fem | 7.75 | 6.48 | 1.19 | .24 |
| Corrected stroke lesion volume | **-15.84** | **-5.13** | **-3.08** | **< .01**** |

*Note.* Isch: ischaemic, fem: female, SpoComp: spoken comprehension, SpoProd: spoken production, WMH CC Fmin: white matter hyperintensities within corpus callosum forceps minor

**5. Excluded neuroimaging variables in stepwise regression Model 1:**

Step 1 SpoComp score

Excluded variables and associated statistics:

| Excluded variables  Stepwise regression | Beta In | t | p |
| --- | --- | --- | --- |
| WMH lesion load CC-Fmaj, sqrt% | .331 | 1.239 | .224 |
| WMH lesion load CC-Body, sqrt% | -.218 | -1.314 | .198 |
| WMH volume, cm^3^ | -.173 | .739 | .465 |
| Stroke lesion load Brocas, sqrt% | .015 | .100 | .921 |
| Stroke lesion load AG + SMG, sqrt% | - .077 | -.522 | .605 |
| Stroke lesion load STG, sqrt% | .021 | .113 | .910 |
| Stroke lesion load Insula, sqrt% | -.299 | -1.724 | .094 |

*Note.* AG+SMG: angular gyrus + supramarginal gyrus, CC-Fmaj: corpus callosum forceps major, CC-Body: corpus callosum body, sqrt: square root, STG: superior temporal gyrus, WMH: white matter hyperintensities

SpoProd score

Step 1 SpoProd score

Excluded variables and associated statistics:

| Excluded variables  Stepwise regression | Beta In | t | p |
| --- | --- | --- | --- |
| WMH lesion load CC-Fmin, sqrt% | -.093 | -.590 | .560 |
| WMH lesion load CC-Fmaj, sqrt% | -.061 | -.389 | .700 |
| WMH lesion load CC-Body, sqrt% | -.229 | -1.489 | .147 |
| WMH volume | -.064 | -.406 | .688 |
| Stroke lesion load Brocas, sqrt% | .105 | .600 | .553 |
| Stroke lesion load AG + SMG, sqrt% | - .310 | -1.900 | .067 |
| Stroke lesion load STG, sqrt% | -.112 | -.520 | .607 |
| Stroke lesion load Insula, sqrt% | -.219 | -1.147 | .260 |

*Note.* AG+SMG: angular gyrus + supramarginal gyrus, CC-Fmin: corpus callosum forceps minor, CC-Fmaj: corpus callosum forceps major, CC-Body: corpus callosum body, sqrt: square root, STG: superior temporal gyrus, WMH: white matter hyperintensities

**6. Linear regression assumptions: Final models tests & plots**

**Model 2 SpoComp score**

Model statistics: R2 = .66, F(5, 31) = 11.98, p < .001). Significant variables: corrected stroke lesion volume, WMH CC-Fmin, stroke type.

**Diagnostic tests:**

Shapiro-Wilk test: *p* = .07

Breusch-Pagan test: *p* = .56

Variance inflation factor: all predictors below 1.1

**
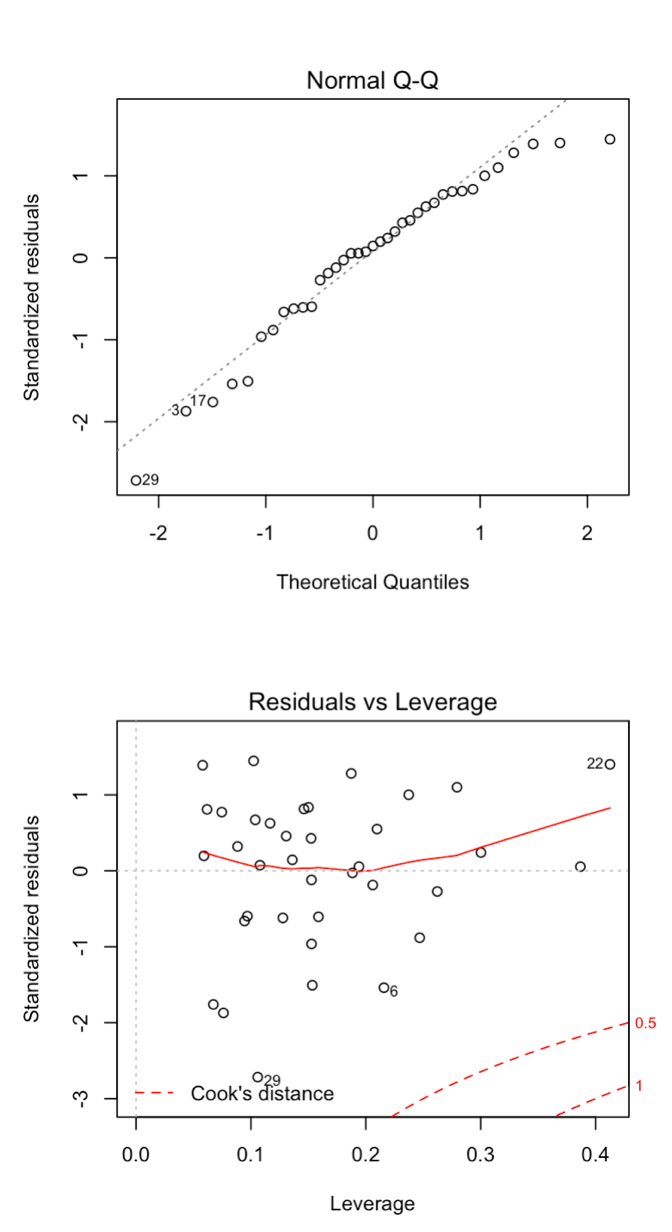
Diagnostic plots:** QQ plot, Cook's distance plot,

**Model 2 SpoProd score**

Model statistics: (R2 = .38, F(4, 29) = 4.60, p = .005). Significant variables: corrected stroke lesion volume.

**Diagnostic tests:**

Shapiro-Wilk test: *p* = .96

Breusch-Pagan test: *p* = .10

Variance inflation factor: all predictors below 1.

**Diagnostic plots:**

QQ plot, Cook's distance plot,


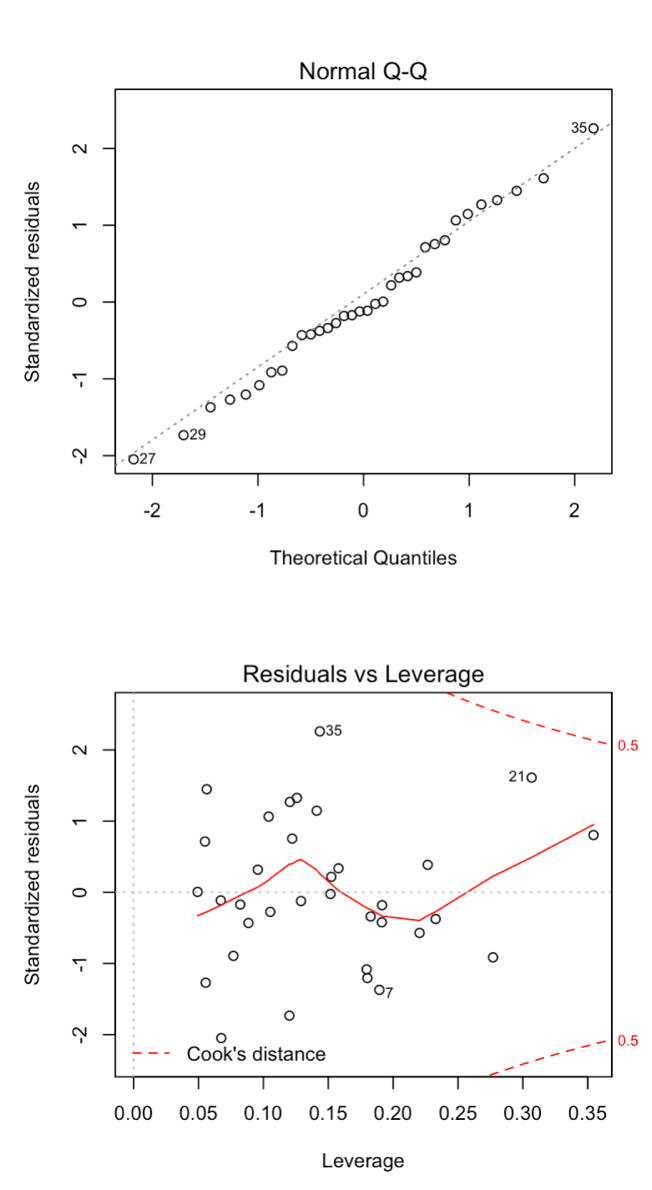

Supplement: Supplementary file 1 — Data S1: Supporting Information. [file HBM-45-e26568-s001.docx]
